# Evaluation of a real-time quantitative PCR to measure the wild *Plasmodium falciparum* infectivity rate in salivary glands of *Anopheles gambiae*

**DOI:** 10.1186/1475-2875-12-224

**Published:** 2013-07-02

**Authors:** Alexandra Marie, Anne Boissière, Majoline Tchioffo Tsapi, Anne Poinsignon, Parfait H Awono-Ambéné, Isabelle Morlais, Franck Remoue, Sylvie Cornelie

**Affiliations:** 1 Laboratoire MIVEGEC (UMR IRD 224 CNRS 5290 UM1-UM2), 911 Av. Agropolis, 34394 Montpellier Cedex 5, France; 2 Laboratoire de Recherche sur le Paludisme, Organisation de Coordination pour la lutte contre les Endémies en Afrique Centrale, BP288 Yaoundé, Cameroon; 3 Centre de Recherche Entomologique de Cotonou, Ministère de la Santé, Cotonou, Bénin

**Keywords:** *Plasmodium falciparum*, *Anopheles gambiae*, Salivary Glands, Quantitative PCR, Multiplex PCR, CSP-ELISA

## Abstract

**Background:**

Evaluation of malaria sporozoite rates in the salivary glands of *Anopheles gambiae* is essential for estimating the number of infective mosquitoes, and consequently, the entomological inoculation rate (EIR). EIR is a key indicator for evaluating the risk of malaria transmission. Although the enzyme-linked immunosorbent assay specific for detecting the circumsporozoite protein (CSP-ELISA) is routinely used in the field, it presents several limitations. A multiplex PCR can also be used to detect the four species of *Plasmodium* in salivary glands. The aim of this study was to evaluate the efficacy of a real-time quantitative PCR in detecting and quantifying wild *Plasmodium falciparum* in the salivary glands of *An. gambiae*.

**Methods:**

*Anopheles gambiae* (n=364) were experimentally infected with blood from *P. falciparum* gametocyte carriers, and *P. falciparum* in the sporozoite stage were detected in salivary glands by using a real-time quantitative PCR (qPCR) assay. The sensitivity and specificity of this qPCR were compared with the multiplex PCR applied from the Padley method. CSP-ELISA was also performed on carcasses of the same mosquitoes.

**Results:**

The prevalence of *P. falciparum* and the intensity of infection were evaluated using qPCR. This method had a limit of detection of six sporozoites per μL based on standard curves. The number of *P. falciparum* genomes in the salivary gland samples reached 9,262 parasites/μL (mean: 254.5; 95% CI: 163.5-345.6). The qPCR showed a similar sensitivity (100%) and a high specificity (60%) compared to the multiplex PCR. The agreement between the two methods was “substantial” (κ = 0.63, P <0.05). The number of *P. falciparum*-positive mosquitoes evaluated with the qPCR (76%), multiplex PCR (59%), and CSP-ELISA (83%) was significantly different (P <0.005).

**Conclusions:**

The qPCR assay can be used to detect *P. falciparum* in salivary glands of *An. gambiae*. The qPCR is highly sensitive and is more specific than multiplex PCR, allowing an accurate measure of infective *An. gambiae*. The results also showed that the CSP-ELISA overestimates the sporozoite rate, detecting sporozoites in the haemolymph in addition to the salivary glands.

## Background

In malaria endemic countries, *Plasmodium falciparum* is transmitted to the human host by the bite from a female *Anopheles* mosquito. *Anopheles gambiaesensu stricto* (*s.s.*) is the most widespread malaria vector throughout the afrotropical belt. In the context of malaria eradication, it is essential for malaria-surveillance programmes to estimate accurately the risk of malaria transmission. Currently, the main indicator of *Plasmodium* transmission is the measure of the entomological inoculation rate (EIR) [1], which is the number of infective mosquito bites per human per night. In field settings, the EIR is commonly estimated by using captured adult mosquitoes. Evaluation of infection prevalence in salivary glands can be measured by counting sporozoites by microscopy [2] or by using the enzyme-linked immunosorbent assay on the head-thorax of the mosquito to detect the surface circumsporozoite protein (CSP-ELISA) [3]. Both methods are known to be labour intensive and it has been shown that CSP-ELISA overestimates the real infection rate by detecting the CSP from the oocysts bursting, two to three days before the sporozoites actually reach the salivary glands [2,4].

Research efforts in recent decades have led to the development of molecular biology tools for detecting *Plasmodium falciparum* in human blood [5] and in mosquito samples [6]. Among these, a multiplex PCR was developed by Padley *et al* to detect the four major species of *Plasmodium* (*P. falciparum*, *Plasmodium malariae*, *Plasmodium ovale,* and *Plasmodium vivax*) in human blood samples [7] and was applied to detect them in *Anophele*s mosquitoes. Multiplex PCR is based on the detection of a Small SubUnit of ribosomal RNA (SSU rRNA) of each *Plasmodium* species but it requires a significant amount of parasite DNA, which is not easily achieved with small tissues like a single pair of salivary glands. Specific and sensitive methods such as quantitative PCR (qPCR) have also been developed to measure the prevalence and intensity of infection in human blood samples [8,9]. In mosquito samples, quantification of *P. falciparum* oocysts in *Anopheles stephensi*[10] and in wild *An. gambiaes.s.*[11] has also been achieved through real-time PCR. The latter study evaluated the difference in susceptibility of malaria infection (oocyst stage) between the M and S molecular form of *An. gambiae s.s.* in Cameroon. In addition, Vernick *et al*[12] estimated the infection prevalence of *P. falciparum* (parasite culture) in *An. gambiae* (insectary-reared mosquitoes) by reverse transcriptase PCR using specific sequences of the Small SubUnit of ribosomal RNA (SSU rRNA) of the sporogonic stages. Recently, a duplex real-time PCR was developed for the detection of the four *Plasmodium* species in field mosquitoes from Benin based on species-specific primers and probes for the gene encoding the small subunit (18S) of *Plasmodium* rRNA [13]. However, in this study, the use of the head-thorax of mosquitoes leads to an inaccurate estimation of the EIR, which should be based only on the sporozoites present in salivary glands.

Therefore, it is important to develop sensitive and rapid diagnostic tools for detecting *Plasmodium* in salivary glands of the *Anopheles* vectors, as this will reveal the true proportion of infective mosquitoes and, consequently, only those that can transmit malaria parasites. The aim of the present study was to evaluate the sensitivity and the specificity of a quantitative PCR method in the detection of wild *P. falciparum* sporozoites in *An. gambiae* salivary glands. First, the qPCR assay based on the mitochondrial cytochrome c oxydase subunit 1 (COX-1) gene described by Boissiere *et al* [11] was tested on infected salivary glands to detect and quantify *P. falciparum*. A comparison of the qPCR method with a multiplex PCR based on the Padley method was also made to identify the most sensitive method. Finally, a comparison of the infectivity rates obtained with these two techniques with those obtained with the CSP-ELISA was performed on the carcasses of mosquitoes without salivary glands. CSP-ELISA was considered the current reference method used in the field. In this paper, experiments were conducted in semi-field conditions. *Anopheles gambiae* mosquitoes were fed on blood from asymptomatic children containing high similar gametocyte densities (from 52.7 to 60.6 gametocytes/μL). In natural settings, mosquito infectivity rate depends on several factors such as gametocyte density, sex ratio and multiclonality of parasites [14-17]. In consequence, this original approach allowed to mimic field conditions, and thereby to evaluate the potential application of this qPCR in field settings. Data showed that qPCR is highly sensitive but more specific than the multiplex PCR. Moreover, this study confirmed that the CPS-ELISA overestimates the infectivity rate by detecting the circulating sporozoites in addition to those present in salivary glands.

## Methods

### Ethics statements

All procedures involving human subjects used in this study were approved by the Cameroonian National Ethical Committee (statement 099/CNE/SE/09). Children identified as gametocyte carriers were enrolled as volunteers after their parents or legal guardians have signed an informed consent form.

### Mosquito collection

The Kisumu strain of *An. gambiae* was provided by the Laboratoire de Lutte contre les Insectes Nuisibles, Institut de Recherche pour le Développement, France. The colony was established and maintained at the insectary in OCEAC (Yaoundé, Cameroon) for the experimental infections. Adult mosquitoes were maintained in standard insectary conditions (27±2°C, 85±5% RH, and 12 h light/dark) and provided with 6% sterile sucrose solution.

### Experimental infections and salivary gland dissection

Female mosquitoes were fed on *P. falciparum* gametocyte carriers. Infectious feeding was performed as previously described [18,19]. Females, three to five days old, were starved for 24 h and allowed to feed on human blood containing *P. falciparum* gametocytes for 35 min. Unfed and partially fed mosquitoes were removed by aspiration and discarded. Fully engorged females were kept in the insectary until dissections 14 days after the infectious blood meal. Mosquitoes were cold-anaesthetized and salivary glands were dissected in 10 μL of buffer containing 7 M urea, 2 M thiourea, and 4% CHAPS (GE, Healthcare). Samples were kept frozen individually at −20°C until processing.

### CSP-ELISA assay

After the dissection of salivary glands, the carcass-thorax-head were tested by ELISA for the presence of *P. falciparum* CSP as described by Burkot and modified by Wirtz *et al*[20]. The monoclonal antibody and positive controls were provided by the Centers for Disease Control and Prevention (CDC, Atlanta, GA, USA). Mosquitoes were considered positive when the optical density (OD) was higher than the mean plus three standard deviations of the negative controls (OD=0.059).

### DNA extraction

DNA extraction from the salivary glands was performed using DNAzol® (Molecular Research Center, Inc, Cincinnati, OH, USA) according to the manufacturer’s instructions. Extracted DNAs were eluted in a final volume of 20 μL water and were stored at –20°C. DNA extraction was checked for the presence of mosquito DNA by specific PCR for *An. gambiae* species [21].

### Identification of *Plasmodium falciparum* by multiplex PCR

The infection status of *P. falciparum* was determined by multiplex PCR as previously described [7] based on the detection of a Small SubUnit of ribosomal RNA of each *Plasmodium* species with five primers: universal reverse *Plasmodium* primer 5′-GTATCTGATCGTCTTCACTCCC-3’, *P. malariae forward* 5′-CGTTAAGAATAAACGCCAAGCG-3′, *P. falciparum forward* 5′- ACAGACGGGTAGTCATGATTGAG-3′, *P. ovale forward* 5′-CTGTTCTTTGCATTCCTTATGC-3′, and *P. vivax forward* 5′-CGGCTTGGAAGTCCTTGT-3′. PCR was performed on 5 μL of eluted DNA with the Taq Hot Start Master mix (Qiagen, Valencia, CA, USA) following the manufacturer’s instructions. PCR amplification was carried out under the following conditions: an initial incubation cycle to activate the enzyme for 45 sec at 95°C followed by 43 cycles of amplification involving 45 sec at 95°C, 90 sec at 60°C and a final extension of 5 min at 72°C.

### Quantitative real-time PCR

qPCR was performed on 1 μL of eluted DNA with the EvaGreen® dye (5X HOT Pol EvaGreen® qPCR Mix Plus (ROX), Euromedex, Souffelweyersheim, France) in the 7300 Real-Time PCR System (Applied Biosystems, Foster City, CA, USA). Specific primers used for the qPCR were 5′-TTACATCAGGAATGTTATTGC-3′ and 5′-ATATTGGATCTCCTGCAAAT-3′ [9,22]. They amplified a 120-bp sequence of the *P. falciparum* cytochrome c oxidase subunit 1 (Cox1) mitochondrial gene. The reaction mixture was prepared following the procedure of Boissière *et al*[11]. Absolute qPCR was performed following the amplification program of an initial melting cycle for 15 min at 95°C followed by 40 amplification cycles at 95°C for 15 sec and 58°C for 30 sec. The melting temperature was determined using a dissociation curve. Curves were generated after amplification: at 95°C for 15 sec (DNA denaturation), at 60°C for 30 sec (double stranded DNA), and at 95°C for 15 sec (single stranded DNA). Fluorescence was monitored allowing the identification of the specific melting point. As described by Boissière *et al*[11], standard curves using 3D7 strain DNA were generated from serial dilution methods and resulting in a quantification range of 6 to 60,000 genomes/μL. These standards were used to determine the concentration of sporozoites in the salivary glands of *An. gambiae*[11].

### Statistical analysis

Statistical analyses were performed using the statistical software R [23], and all differences were considered significant at P values of <0.05. The means of the amplification efficiencies between the standard samples and the salivary gland samples were compared using the Mann-Whitney-Wilcoxon test. Cohen’s kappa co-efficient (κ) was calculated to measure the agreement between the qPCR and multiplex PCR. Methods were compared using the McNemar test.

## Results

**Figure 1:**
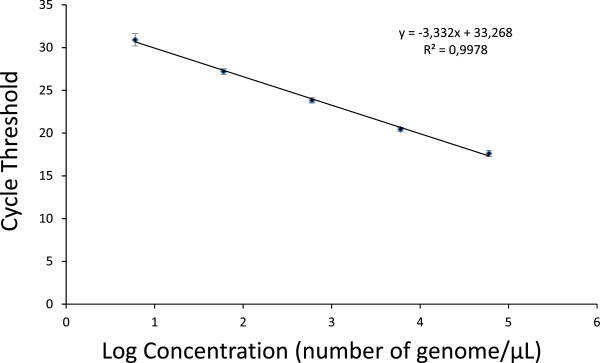
**Standard curve of qPCR using serial dilutions of DNA from cultured parasites.** Calibration curve was generated using 53 calibrations curves. The curve is based on the known DNA concentration (genomes/μL) and shows the reproducibility. Error bars show the standard deviation for each DNA standard from 6 to 60, 000 genomes/μL [11]. © Boissiere *et al*[11].

The detection threshold of the method was determined by using a five-fold serial dilution of genomic DNA isolated from a 3D7 culture of *P. falciparum,* allowing a quantification range from 6 to 60,000 genomes/μL, as previously described [11]. The reproducibility of the test was confirmed by using a composite of 53 standard curves showing a standard deviation <0.75 and a regression value equal to 0.998 for the five data points (Figure 1). The means of the amplification efficiencies per amplicon for the cultured parasites and the salivary gland samples were 94.3% (± 0.6) and 94.1% (± 0.3), respectively, and the difference was not significant (P=0.065). This result showed that the prevalence and intensity of infection can be evaluated using this method. Absolute quantification of *P. falciparum* genomes in *Anopheles* salivary gland samples was based on the calibration curve (composite of the 53 standard curves) with a detection limit of six genomes/μL (120 sporozoites by pair of salivary glands in this study). *Plasmodium falciparum* parasitaemia in salivary gland samples reached 9,262 parasites/μL, with an infection mean of 254.5 parasites/μL (95% CI: 163.5-345.6). This result showed an heterogeneity in the *Plasmodium* infection intensity among mosquitoes, as observed in the salivary glands. For the first time, the optimized qPCR enabled specific detection and quantification of total *Plasmodium* parasitaemia (genome/μL) in *An. gambiae* salivary glands.

The prevalence of *P. falciparum* was assessed on DNA extracted from the salivary glands of 364 *An. gambiae* using qPCR and multiplex PCR. The qPCR revealed 276 positive (76%) and 88 negative (24%) salivary glands for *P. falciparum,* whereas the presence of *Plasmodium* DNA was found in 217 (60%) salivary glands by multiplex PCR (Table 1). The statistical analysis showed that differences obtained by both methods were significant (McNemar test: multiplex PCR *vs* qPCR, P <0.001). The 217 positive salivary glands with multiplex PCR were confirmed as positive with qPCR. Among the 147 negative salivary glands detected with multiplex PCR, 88 were also negative with qPCR and 59 were identified as positive. The qPCR method presented high values of sensitivity of 100% (Se = (217/217)*100) and specificity of 60% (Sp = (88/147)*100) when compared to the multiplex PCR, considered here as the reference test. The agreement between qPCR and multiplex PCR was “substantial” (κ = 0.63 and P <0.001). In the field and especially in the context of malaria eradication or low transmission, the number of sporozoites in salivary glands could be very low and multiplex PCR may not be sensitive enough to detect low infection rates. Furthermore, this method requires a high amount of DNA template and therefore it seems unsuitable for investigating infection in mosquito salivary glands. This may explain the low positivity rate detected in the present study (59%). Moreover in *Plasmodium spp*, Cox1 mitochondrial gene is present in higher quantity than the SSU rRNA gene. Indeed, mitochondrial DNA is composed to approximately 20 copies per cell [24], whereas the SSU rRNA genes are presented in 4-8 copies [25]. Consequently, the use of the Cox1 gene for the qPCR increases its specificity. qPCR, known for its sensitivity and for the small amount of DNA required, thus seemed a feasible way to detect *Plasmodium* in *Anopheles* salivary glands. Using this technique, a 1.25-fold higher prevalence rate of *P. falciparum* infection compared to the multiplex PCR and a detection limit of six sporozoites/μL were observed. Interestingly, false-negative samples determined by multiplex PCR were detected as positive using qPCR. In addition, this method estimates the *Plasmodium* intensity level in contrast to multiplex PCR, even in very small biological samples like the single pair of salivary glands used here. This method could open the way for determining the relationship between the sporozoite load in salivary glands and the infectiousness of the *Anophele*s mosquito. However, this qPCR approach identified only *P. falciparum* species, in contrast to multiplex PCR. Although *P*. *falciparum* is the species causing the majority of clinical cases of malaria in Africa, a recent study in rural Benin has shown that *P. faciparum* accounted for 91% of the malaria infections, evaluated by thick blood smears. Mixed infections with *P. malariae* or *P. ovale* were also detected at 3% and 2% of the tested slides, respectively [26]. A multiplex qPCR was developed to discriminate the four species of *Plasmodium* in human blood samples [27,28], and one was developed very recently by Sandeu *et al* in mosquitoes [13]. However, the latter method was performed and optimized using a duplex qPCR on the head-thorax of mosquitoes, consequently using DNA from both the circulating sporozoites and those in the salivary glands [13].

**Table 1:** **Comparison of qPCR and multiplex PCR techniques for detection of *Plasmodium falciparum *sporozoites in salivary glands of *Anopheles gambiae***

**Salivary glands samples**	**qPCR positive**	**qPCR negative**	**Total**	**Sensitivity**	**Specificity**	**Kappa (κ)**
**PCR positive**	217	0	217 (60%)	100%	60%	0.63
**PCR negative**	59	88	147 (40%)			
**Total**	276 (76%)	88 (24%)	364 (100%)			

McNemar test: qPCR *vs* multiplex PCR, *P* <0.001.

Some multiplex qPCR assays have also used the EvaGreen® dye, as it was done in the present study [29,30]. Therefore, it seems possible to adapt the present qPCR method so as to carry out multiplex qPCR detection of the four species of *Plasmodium*. Evagreen® dye is a DNA-binding dye with many features that make it superior to the SYBR® Green I for qPCR [29,31]. Furthermore, this dye is compatible with all common real-time PCR cyclers [32] and is currently about half the price (€0.16 per reaction) of the SYBR® Green (€0.53 per reaction) commonly used. The duplex qPCR performed by Sandeu *et al* used the Taqman technique (€1.12 per reaction). In conclusion, the qPCR developed here is cost-effective and therefore suitable for large field studies. It is also cheaper than the multiplex PCR (€1.60 per reaction).

Detection of the presence of the *Plasmodium* parasite by CSP-ELISA was also tested on the head-thorax carcasses of the same mosquitoes without salivary glands, thus detecting only circulating sporozoites. A total of 302 mosquitoes were found to be *P. falciparum* positive (range OD: from 0.164 to 2.420) (Table 2). The results of the CSP-ELISA showed that a higher number of positive mosquitoes (83%) were detected compared to multiplex PCR (60%) and qPCR (76%). The statistical analysis revealed statistically significant differences between the three methods (McNemar test: CSP-ELISA *vs* qPCR, P <0.001; CSP-ELISA *vs* PCR, P <0.001) (Table 2). Of the 302 *Plasmodium*-infected head-thorax-carcasse samples, 261 were found to be positive while 41 samples were negative using the qPCR method. According to these results, 11.2% of the mosquitoes were found to be *Plasmodium* positive in the head-thorax-carcasses but not in the salivary glands, meaning that circulating sporozoites can be detected using CSP-ELISA even in non-infective mosquito. This finding is in accordance with other studies [2,33,34] showing that the CSP-ELISA assay (performed on head-thorax including salivary gland) overestimates the sporozoite rate in mosquitoes by detecting circulating sporozoites. Indeed, parasites covered by CSP, spread into the haemolymph for two to three days before they reach the salivary glands [35]. Moreover, it has been shown that only 10-20% of sporozoites reach the salivary glands [36-38] and that some mosquitoes could be refractory to the entrance of sporozoites in salivary glands [39]. Consequently, CSP-ELISA, which is routinely used in the field, detects infected mosquitoes but not necessarily the infective ones.

**Table 2:** **Comparison of CSP-ELISA with qPCR and multiplex PCR for detection of *Plasmodium falciparum *sporozoites**

	**CSP-ELISA positive**	**CSP-ELISA negative**	**Total**
**qPCR positive**	261	15	276 (76%)
**qPCR negative**	41	47	88 (24%)
**Total**	302 (83%)	62 (17%)	364 (100%)
**PCR positive**	211	6	217 (60%)
**PCR negative**	91	56	147 (40%)
**Total**	302 (83%)	62 (17%)	364 (100%)

McNemar test: CSP-ELISA *vs* qPCR, *P* <0.001; CSP-ELISA *vs* PCR, *P* <0.001.

CSP-ELISA was performed on head-thorax-carcasses, and qPCR and multiplex PCR were performed on salivary glands DNA.

## Conclusion

Estimation of malaria transmission requires sensitive and specific tools for the evaluation of infective mosquitoes, i e, detection of sporozoites in *Anopheles* salivary glands. This study showed that real-time quantitative PCR can be used to detect and quantify sporozoites of wild *P. falciparum* in the salivary glands of *An. gambiae.* This qPCR can be performed on small samples such as the DNA of *P. falciparum* sporozoites extracted from a single pair of salivary glands of *An. gambiae* with a sensitivity of six genomes/μL. In the present study, the real-time quantitative PCR was compared for the first time with multiplex PCR and CSP-ELISA methods.

qPCR is highly sensitive but more specific than multiplex PCR. Moreover, qPCR with EvaGreen® dye is reliable, reproducible, and cost-effective. This method is feasible for evaluating the *P. falciparum* infection rate in the salivary glands and it can lead to an accurate estimation of the risk of transmission in field settings, which were overestimated by CSP-ELISA. Improving the estimation of the EIR with this method could have significant implications on vector control strategies and on the evaluation of their effectiveness.

## Competing interests

The authors declare that they have no competing interests.

## Authors’ contributions

AM, AB, IM and SC conceived and designed the experiments. AM, AB, MTT and IM carried out the experiments. AM and AB analysed the data. FR, PHAA and IM contributed reagents/materials/analysis tools. AM, AB, AP and SC wrote the paper. All authors read and approved the final manuscript.
